# Imaging endpoints for clinical trials in Alzheimer’s disease

**DOI:** 10.1186/s13195-014-0087-9

**Published:** 2014-12-20

**Authors:** David M Cash, Jonathan D Rohrer, Natalie S Ryan, Sebastien Ourselin, Nick C Fox

**Affiliations:** Dementia Research Centre, Box 16, The National Hospital for Neurology and Neurosurgery, Queen Square, London, WC1N 3BG UK; Translational Imaging Group, Centre for Medical Image Computing, University College of London, 3rd Floor, Wolfson House, 4 Stephenson Way, London, NW1 2HE UK

## Abstract

As the need to develop a successful disease-modifying treatment for Alzheimer’s disease (AD) becomes more urgent, imaging is increasingly used in therapeutic trials. We provide an overview of how the different imaging modalities are used in AD studies and the current regulatory guidelines for their use in clinical trials as endpoints. We review the current literature for results of imaging endpoints of efficacy and safety in published clinical trials. We start with trials in mild to moderate AD, where imaging (largely magnetic resonance imaging (MRI)) has long played a role in inclusion and exclusion criteria; more recently, MRI has been used to identify adverse events and to measure rates of brain atrophy. The advent of amyloid imaging using positron emission tomography has led to trials incorporating amyloid measurements as endpoints and incidentally to the recognition of the high proportion of amyloid-negative individuals that may be recruited into these trials. Ongoing and planned trials now commonly include multimodality imaging: amyloid positron emission tomography, MRI and other modalities. At the same time, the failure of recent large profile trials in mild to moderate AD together with the realisation that there is a long prodromal period to AD has driven a push to move studies to earlier in the disease. Imaging has particularly important roles, alongside other biomarkers, in assessing efficacy because conventional clinical outcomes may have limited ability to detect treatment effects in these early stages.

**Electronic supplementary material:**

The online version of this article (doi:10.1186/s13195-014-0087-9) contains supplementary material, which is available to authorized users.

## Introduction

Treatments for Alzheimer’s disease (AD) and related disorders are currently limited to those that provide only modest symptomatic benefit. Disease-modifying therapies are urgently needed, especially those that would delay the onset of clinical decline. An effective treatment that delays symptom onset by 5 years has been estimated to potentially reduce predicted dementia prevalence and healthcare costs by 40 to 50% [1]. A large number of candidate disease-modifying therapies are under development [2]; the studies that will be assessing these therapies are increasingly incorporating a range of imaging and other biomarkers to understand better their effects and to show evidence of disease slowing. This evidence is particularly important for guiding decisions about which therapies to take forward into large and expensive late-phase trials.

Imaging endpoints provide at least three possible benefits to clinical trials in dementia. First, they provide a means of assessing potential disease-modifying effects and differentiating these from symptomatic benefits that do not affect underlying pathological progression. Many imaging biomarkers have been shown to correlate with disease severity, as well as predict future progression in subjects yet to show clinical symptoms. Second, the quantitative nature of the imaging biomarkers often have far less variability than the primary cognitive and functional endpoints, and thus will require smaller sample sizes to be powered to show a statistically significant effect. These quantitative endpoints are objective measures where the data can be saved for further re-analysis, while assessments of clinical status are more subjective and cannot be revisited at a later stage. Finally, imaging can be used in assessing the safety of a treatment, potentially identifying adverse effects before symptoms are reported by patients.

This article provides an overview of how imaging has been used as an endpoint in clinical trials in AD. We assessed published studies and also the controlled clinical trials database ClinicalTrials.gov. This review includes trials in mild to moderate AD, newer trials involving patients with mild cognitive impairment (MCI), and trials aimed at (secondary) prevention to slow the onset of clinical AD in preclinical populations. In more recent trials and in those with a focus earlier in the disease, study designs rely more on imaging to select populations and to help assess safety and efficacy, although imaging still provides secondary or exploratory endpoints. Finally, we describe the regulatory guidance on how these biomarkers should be used in trials.

## Review

### Imaging biomarkers in Alzheimer’s disease

The most commonly used imaging modality in the study of AD has been volumetric T1-weighted magnetic resonance imaging (MRI). These images provide high-resolution (~1 mm) structural images with good tissue contrast. Longitudinal natural history cohort studies have demonstrated changes in global measures based on T1 images, such as whole brain volume or ventricular volume, as well as regional measures, particularly the hippocampus, that are several times higher in AD patients than in age-matched cognitively intact individuals. These studies have typically shown greater effect sizes and therefore lower samples sizes for imaging when compared with clinical endpoints.

In a 38-centre imaging continuation of a therapeutic trial of milameline [3], the estimated number of subjects per arm required to detect a 50% reduction in the rate of decline over 1 year was only 21 for hippocampal volume compared with 320 for the Alzheimer’s Disease Assessment Scale – cognitive subscale and 241 for the Mini Mental State Examination. Similar improvements in sample sizes needed to power for a reasonable treatment effect were observed in the large Alzheimer’s Disease Neuroimaging Initiative study, where numerous studies using different atrophy measurement techniques have produced sample size estimates in the order of 100 to 200 per arm needed for an AD trial with 80% power to detect a 25% improvement in annual rate of decline [4-6]. Note that this 25% improvement in an imaging biomarker may not relate to a 25% improvement in clinical measures. A complete review of sample size estimates from the Alzheimer’s Disease Neuroimaging Initiative can be found in [7]. Similar sample size estimates for a MCI trial would be higher, in the order of 300 to 600 per arm, and very dependent on inclusion criteria. Such studies led to the increased inclusion of volumetric measures as endpoints in clinical trials.

As with volumetric MRI, fludeoxyglucose (FDG)-based positron emission tomography (PET) has been extensively investigated in natural history studies of AD, revealing characteristic and progressive reductions in regional measurements of the cerebral metabolic rate for glucose, particularly involving the posterior cingulate, parietal and temporal regions. The statistical power of FDG-PET to detect the ability of a putative disease-modifying therapy to slow rates of regional decline in randomised clinical trials has been estimated, with the number of AD patients per treatment arm needed to detect an effect with FDG-PET being either greater than or similar to that needed with MRI [8], roughly 200 per arm for AD [9].

Another family of PET radiotracers that shows great utility for AD research is the ligands that bind to fibrillar forms of amyloid beta. The initial amyloid PET imaging studies were performed using the carbon-11-based ligand Pittsburgh compound B (PIB). These studies, along with data from cerebrospinal fluid (CSF) measurements of amyloid beta 1-42, provided further evidence that the disease process begins years before symptoms are observed clinically. These measures of amyloid burden provided *in vivo* support for a clinical diagnosis of AD. Due to the short (20 minutes) half-life of carbon-11, multicentre studies using this tracer can be challenging. A number of fluorine-18-based amyloid tracers have since been developed; due to the longer (110 minutes) half-life, using these tracers does not require a cyclotron on site. There is currently limited information regarding sample size estimates for amyloid imaging.

An alternative to amyloid imaging is CSF biomarkers, with a low CSF amyloid beta 1-42 level having similar sensitivity for cerebral amyloid deposition. CSF examinations do not provide the ability to quantify regional deposition, but they are more readily available and do allow for assessment of other markers of pathology within a single (admittedly invasive) assessment: CSF total tau and phosphor-tau being important markers of neurodegeneration. In terms of application to clinical trials, CSF biomarkers can be either complementary or an alternative to amyloid imaging depending on use. There is also evidence that the two measures might be interchangeable for the purpose of inclusion criteria for some trials, as there is good agreement between CSF and PET measures, which have a strong inverse correlation [10-12]. For more information on the utility of CSF biomarkers, the authors would refer the readers to the review article by Blennow and colleagues [13].

While the previous imaging modalities are used for biomarkers of efficacy, MRI scans can also be used for safety endpoints in trials. In particular in some anti-amyloid immunotherapy trials, a number of patients developed side effects associated with what has been termed amyloid-related imaging abnormalities (ARIA). Two major types of ARIA have been described: ARIA-E, signal hyperintensities seen on T2-weighted fluid-attenuated inversion recovery magnetic resonance sequences felt to represent vasogenic oedema and/or sulcal effusion; and ARIA-H, signal hypointensities on T2*-weighted gradient recalled echo magnetic resonance sequences that are thought to represent haemosiderin deposits including microhaemorrhages and superficial siderosis [14]. As these safety assessments have become an essential aspect of clinical trials in AD, a rating scale has been developed to help standardise these measures [15].

### Search strategy

The data search was completed on 15 May 2014 using the PubMed and ClinicalTrials.gov databases.

The PubMed search was used to find all published results from completed clinical trials where imaging was used as an endpoint. For the PubMed search, two Medical Subject Headings terms were used for the disease: ‘Alzheimer disease/drug therapy’ or ‘Mild Cognitive Impairment/drug therapy’. These were combined with the following outcome-related Medical Subject Headings terms: ‘Biological Markers/analysis’, ‘Biological Markers/drug effects’, ‘magnetic resonance imaging’ and ‘positron emission tomography’. Only publications tagged as a Clinical Trial Medical Subject Headings publication type were considered. The resulting literature search was limited to the last 10 years of publication date and all articles were reviewed for relevance, including only publications where imaging was used as an endpoint for the study. Imaging studies that used voxel-wise analysis with no specific measurable quantity were excluded because they would not be suitable as an endpoint for a trial.

The search on ClinicalTrials.gov was used to determine those completed and currently active clinical trials in AD and MCI in which imaging was an endpoint listed in the trial entry. ‘Alzheimer disease’ and ‘mild cognitive impairment’ were used as the search terms in the condition field, and the following search terms were used for the outcomes: ‘MRI’, ‘PET’, ‘magnetic resonance imaging’, ‘positron emission tomography’, ‘hippocampal volume’, ‘brain volume’ and ‘brain atrophy’ – only trials active within the last 10 years were considered. The resulting trial entries were also reviewed for relevance. The results from both searches were combined into Additional file 1.

### Mild to moderate Alzheimer’s disease

Most of the reviewed trials in dementia have enrolled a population diagnosed with probable AD according to a standard diagnostic criterion, such as that of the National Institute of Neurological and Communicative Disorders and Stroke–Alzheimer’s Disease and Related Disorders Association working group [16]. Most studies recruit patients either with mild or mild/moderate severity, primarily determined by a range of Mini Mental State Examination scores.

#### Magnetic resonance imaging as a safety endpoint

Although imaging has long been used to assess adverse events in trials in AD, this was largely in the investigation of a symptomatic event (for example, a cerebrovascular event). More recently the introduction of very biologically active therapies for AD has led to increased use of MRI as a proactive safety assessment. One of the drivers for the increased use of MRI as a safety endpoint was the (interrupted) trial of active anti-amyloid vaccination (AN-1792), where there were reports of meningoencephalitis in ~6% (18/300) of immunised participants in the treatment arm [17,18]. Active vaccination with the CAD106 vaccine had three adverse events out of 46 participants based on MRI findings, although none were considered serious or associated with the central nervous system changes that suggested meningitis or encephalitis [19].

Subsequent to the AN1792 study, the majority of immunotherapy trials have used passive rather than active immunotherapy, and imaging is now necessary as a safety endpoint due to the risk of ARIA. ARIA was observed in a phase I study for bapineuzumab [20] and in subsequent phase II [21] and phase III [22] studies, where dose and the presence of an ε4 allele of the apolipoprotein E (ApoE) gene appeared to be risk factors [23]. ARIA-E findings have also been reported in subjects from a phase II study of gantenerumab [24] and a phase II study of the gamma secretase inhibitor avegacestat [25]. Trials of two monoclonal antibodies, ponezumab [26,27] and solanezumab [28,29], the nonselective gamma secretase inhibitor semagacesat [30], the anti-amyloid aggregation agent scyllo-inositol [31] and the intravenous immunoglobulin administration Octagam [32] have used MRI for safety endpoints and showed no significant treatment-related findings of ARIA.

#### Structural magnetic resonance imaging as an efficacy endpoint

Numerous clinical trials of a wide range of compounds in AD have reported imaging endpoints based on structural MRI. A summary of findings is presented in Table 1. Numerous studies showed no significant treatment effects of any atrophy-related outcome measure: docosahexaenoic acid [33], intravenous immunoglobulin [32] and rosiglitazone [34]. Given the sample size estimates from the Alzheimer’s Disease Neuroimaging Initiative discussed previously, it could be argued that many of these studies were underpowered for the atrophy measure. The recent high-profile phase III studies involving semagacestat [30] and solaneuzumab [29] also showed no statistically significant effect of treatment. In some cases, a treatment effect opposite to the expected direction – a phenomenon that has been referred to as paradoxical volume loss – was observed.

**Table 1:** **Published results of clinical trials in mild to moderate Alzheimer’s disease where volumetric magnetic resonance imaging was used as an imaging endpoint**

**Compound**	**Subject numbers**		**Follow-up duration (months)**	**Treatment effect**
	**Placebo arm**	**Treatment arm**		
AN-1792 [35]	57	231^a^	11	Ventricles (⇑), whole brain atrophy (⇑, antibody responders only), hippocampus (⇔)
Atorvastatin and donepezil [38]	64^b^		18	Whole brain (⇔), hippocampus (⇓)
Bapineuzumab (phase II) [21]	122	107	18	Whole brain (⇓, in ε4 noncarriers)^c^, ventricles (⇑, in ε4 carriers)^d^
Bapineuzumab (phase III) ε4 carriers [22]	238	352	18	Whole brain (⇔)
Bapineuzumab (phase III) ε4 noncarriers [22]	244	315^e^	18	Whole brain (⇔)
CAD106 [19]	7/5	24/21^f^	6, 12	Whole brain (⇔)^g^, ventricles (⇔), hippocampus (⇓)^h^
Docosahexaenoic acid [33]	49	53	18	Whole brain (⇔), ventricles (⇔), hippocampus (⇔)
Intravenous immunoglobulin [32]	7	21	3, 6	Whole brain (⇔), hippocampus (⇔)
Memantine [40]	40^i^		12	Whole brain (⇔), ventricles (⇔), hippocampus(⇓, right only)
Memantine [41]	118	110	12	Whole brain (⇔), hippocampus (⇔)
Rosiglitazone [34]	38	38	6, 12	Whole brain (⇔)
Scyllo-inositol [31]	83	259	18	Whole brain (⇔), ventricles (⇑), hippocampi (⇔)
Semagacestat [30]	208^b^		18	Whole brain (⇔), hippocampus (⇔)
Solaneuzumab [29]	370/400^j^	370/406	18	Whole brain (⇔), hippocampus (⇔)
Tramiprosate [45,46]	109	203^k^	18	Hippocampus (⇓)^l^

⇑, Treatment effect of increased atrophy (or ventricular enlargement); ⇓, treatment effect of decreased atrophy (or ventricular enlargement); ⇔, no treatment effect found. ^a^Of these 231 subjects in the treatment arm, 45 were antibody responders. ^b^The number of subjects enrolled in the magnetic resonance imaging substudy does not identify a division between the placebo arm and the treatment arm, thus the number in the cell indicates the total number of subjects over both arms. ^c^Less brain atrophy (10.7 ml over 71-week follow-up) in apolipoprotein E noncarriers receiving bapineuzumab. ^d^More ventricular enlargement (2.6 ml over 71 week follow-up) in apolipoprotein E carriers receiving bapineuzumab. ^e^The 315 subjects consisted of 169 at 0.5 mg/kg dose and 146 at 1.0 mg/kg dose. ^f^The CAD106 study had two cohorts, where the treatment arm dosage was different (Cohort I, 50 μg; Cohort II, 150 μg). ^g^One global measure of atrophy, left cerebral white matter, did show a treatment effect in Cohort I, but this did not survive correction for multiple comparisons. ^h^In Cohort I, the right hippocampus showed a treatment effect and left hippocampus showed a trend towards treatment effects, but these did not survive multiple comparisons. ^i^Single-group open-label study where subjects had a 24-week lead-in period, followed by 24 weeks treatment of memantine. ^j^The publication consisted of two phase III studies, neither of which showed any treatment effects. ^k^Two treatment arms: 103 subjects at 100 mg twice daily, 100 subjects at 150 mg twice daily. ^l^Original model showed no treatment effects, but *post-hoc* analysis putting in site as a random effect and important covariates showed a treatment effect.

The most commonly used atrophy measure in clinical trials is a global measure of whole brain atrophy. In the AN-1792 study, unexpected (or paradoxical) increased brain volume loss was observed in the antibody responders and there was a strong association between antibody titre and brain volume loss: subjects who generated the higher titres of antibodies had greater volume loss [35]. In a very small subset of study participants who underwent longer term follow-up scans 4.5 years after baseline, this increase in atrophy was no longer present [36]. While a phase II study of bapineuzumab showed no treatment effect in whole brain atrophy, there was a substantial treatment effect (10.7 ml/year) observed when restricting the analysis to APOE ε4 noncarriers only [21]. This finding was not replicated in the larger phase III study of noncarriers [22].

Ventricular enlargement can be a sensitive (although nonspecific) volumetric measure in dementia. Increased ventricular expansion was seen in the AN-1792 study with some suggestion of greater ventricular expansion relative to global brain loss. A similar finding was also observed in a phase II study of scyllo-inositol, although the finding (3.2 ml/year increase, *P* = 0.049) was not corrected for multiple comparisons and no other measures (whole brain, hippocampus and cortical thickness) showed any treatment effects [31]. The phase II study of bapineuzumab showed greater ventricular enlargement, but in ε4 carriers only [21]. This was also found in the two phase III studies, although the increase was much smaller [37].

Measures of medial temporal lobe atrophy (hippocampi, entorhinal cortex) are far more specific to AD. In the AN1792 study, antibody responders also exhibited increased hippocampal atrophy, but this finding was not statistically significant. In one of the cohorts of the CAD106 vaccine, a slowing of hippocampal atrophy was seen in one of the cohorts, but this did not survive correction for multiple comparisons. Two studies of atorvastatin both observed treatment effects of reduced hippocampal atrophy, albeit with caveats. In the LEADe study [38], there were significant baseline differences in demographics as well as the Alzheimer's Disease Cooperative Study – Clinical Global Impression of Change score, which was one of the co-primary outcomes. In the Alzheimer’s Disease Cholesterol Lowering trial this finding did not reach statistical significance and was based primarily on right hippocampal volume [39].

In a phase IV, open-label single-group study of memantine where the pretreatment and post-treatment rates of atrophy were compared, a treatment effect was observed in right hippocampal atrophy, although all other measures (whole brain, ventricular, left hippocampal) showed no treatment effects [40]. This was not confirmed in a larger, multicentre double-blinded and placebo-controlled trial [41], leading to the possibility that this surprising finding in the open-label study might be a false positive. This concern is supported by the fact that the pretreatment rate of right hippocampal atrophy (10.8%) is much larger than the 4 to 5% typically seen in most AD studies. Atrophy rates of this magnitude would be outside what would normally be expected and would not be compatible with cross-sectional results [42]. Hippocampal asymmetry in AD is an ongoing research area [43,44], with different findings possibly due to different segmentation protocols and algorithms. In the open-label study, additional *post-hoc* analysis was performed to test this finding, including the removal of outliers, and the significant decrease in right hippocampal atrophy was still present.

The unexpected findings of increased brain volume loss (and/or ventricular expansion) have been of great interest in the community and will probably impact the design of upcoming trials. These findings occur across multiple studies, although they often do not quite reach statistical significance, as these effect sizes have been relatively small. The strongest evidence for these ‘counterintuitive’ treatment-related effects is in increased ventricular expansion, which has the most sensitivity but least specificity to AD. One of the most important unanswered questions around these findings is whether this is a transient effect, as there are not enough long-term follow-up data to determine whether atrophy later slows or remains increased compared with placebo.

There are numerous important aspects to consider with regards to trial design for volumetric magnetic resonance analysis. The first is what type of measure is going to be used. Manual measures require expert training, but can still result in high variability in the measurement, especially in structures such as the hippocampi. Automated measures help reduce this variability, especially with constraints built in to enforce consistency longitudinally, but these methods could introduce bias. Another critical decision is which statistical analysis to use in the plan, as can be illustrated by the results from the multicenter phase III Alphase study on tramiprosate. The initial model, as specified in the trial protocol, resulted in a treatment effect of increased hippocampal atrophy over a 78-week duration. However, this model indicated very strong site effects, and a *post-hoc* analysis model including key covariates showed a treatment effect in the opposite, expected direction of decreased atrophy [45,46].

#### Positron emission tomography imaging of amyloid deposition

Amyloid imaging provides an attractive option to show efficacy in therapies that target removal of amyloid. A PIB substudy of a phase II bapineuzumab trial acquired data at two expert PET centres. The bapineuzumab arm had a reduction in the standard uptake value ratio of 0.09 compared with the placebo arm, which showed a 0.15 increase [47]. These results were not replicated in phase III studies, which enrolled a larger number of subjects from a larger number of sites. While there was still a significant difference in the carrier trial, it was a smaller effect than in the phase II study. In the noncarriers there was no difference in the standard uptake value ratio due to treatment [22]. Unlike other published studies, the bapineuzumab trials contained eligibility criteria requiring evidence of amyloid positivity on the baseline PIB scan. As a result, 15% from the phase II study and 6.5% of carriers and (a surprisingly high) 36% of the noncarriers from the phase III study were below the specified threshold and were excluded.

Another study on phenserine showed no significant change in amyloid deposition from baseline at 3 months or 6 months [48]. Results from a gantenerumab study at three PET sites also showed some evidence of amyloid removal. The placebo group had a 20% increase in the standard uptake value ratio from baseline to end of treatment, while the lower dose of treatment exhibited only a slight increase of 5% and the high dose showed a decrease in the standard uptake value ratio of 15% [24]. Current phase III studies of gantenerumab in AD are ongoing. Two large phase III clinical trials have used the fluorinated tracer AV-45 in substudies. Both semagacestat and solaneuzumab showed no treatment effects on amyloid deposition [29,30]. As amyloid imaging is used in larger studies with more sites, quality control and assurance procedures will become critical to remove sources of variability that could mask a true treatment effect.

#### Positron emission tomography imaging of glucose metabolism

FDG-PET has not been used as frequently as structural MRI or amyloid PET in large multicentre trials. For a more thorough review on FDG use in multicentre clinical trials in dementia, see the review by Herholz and colleagues [49]. One of the most promising signals from that review was on the pilot study involving intranasal insulin, where hypometabolism was reduced in subjects with AD [50]. Based in part on the results of that study, a larger phase II/III study was launched and is ongoing at the time of writing. In addition to the studies reported in the review, there were no treatment effects on FDG in the phase III semagacestat study.

### Prodromal Alzheimer’s disease/mild cognitive impairment trials

Recent clinical trials in symptomatic patients with probable AD have failed to show substantial evidence of clinical benefit, but there have been observations of decreased amyloid burden at autopsy or on amyloid imaging in treated patients. These results have fostered the view that clinically meaningful disease modification may be possible if treatment begins at an earlier part of the disease process, because intervention may be too late once downstream neurodegeneration has become established. A number of clinical trials have enrolled individuals who do not fulfil criteria for dementia but are thought to have AD as the underlying cause for their MCI, some of which are simultaneously enrolling trials in both mild to moderate AD and MCI. New diagnostic criteria permit a diagnosis of AD before dementia [51-53], with a growing emphasis on the use of biomarkers to support the diagnosis. In these trials, imaging will not only play a role as endpoints, but also in the inclusion/exclusion criteria. Enrichment of trials using imaging biomarkers is gaining acceptance; in 2011, the European Medical Agency issued a favourable opinion on qualifying hippocampal volume as a method of enrichment for prodromal AD trials [54]. Reducing heterogeneity of the population in terms of key biomarkers could provide the needed power to make these studies feasible and avoid situations where changes in amyloid are being measured on trial participants, a subset of which are amyloid-negative.

A few trials in MCI with imaging endpoints have completed and published their results. One of the earliest was the InDDEx study, which tested whether rivastigmine delayed the onset of AD. The results showed a treatment effect of reduction in ventricular enlargement for rivastigmine at 12 and 24 months but not at the end of treatment, and these findings did not survive correction for multiple comparisons [55]. Two studies investigated the effect of donepezil: an Alzheimer’s Disease Cooperative study on donepezil and vitamin E, which showed no treatment effects, but a trend towards slowing hippocampal atrophy in the APOE ε4 carriers [56]; and a substudy from another donepezil trial where no effects on hippocampal or entorhinal cortex atrophy were found [57]. However, this latter study, which included more subjects but involved a shorter follow-up duration, did find treatment effects for whole brain volume, ventricular enlargement and cortical grey matter volume. Finally, a UK study using B vitamins showed a significant treatment effect of a 30% reduction in the rate of brain atrophy [58]. These results require confirmation but may suggest that treatment earlier in the disease may be more likely to show effects on imaging endpoints.

### Preclinical prevention trials

In recent years there has been growing interest in beginning treatment even earlier than MCI/prodromal AD. At the MCI stage, the presence of cognitive deficits and imaging changes such as hippocampal atrophy suggest that a considerable burden of pathology has already become established. Thus, it may be necessary to intervene earlier in the pathological cascade to prevent the development of downstream, irreversible changes. This approach would be more analogous to successful preclinical studies of amyloid-modifying therapies in transgenic animal models, where treatment is introduced when amyloid pathology is minimal and there is no neurodegenerative phenotype [59].

The main challenge in prevention studies is to identify cognitively normal individuals who are already accumulating AD pathology. Frameworks for conceptualising and defining the preclinical stage of AD have been developed that will aid in properly selecting patients for these studies [60,61]. These frameworks are heavily reliant on imaging endpoints, as multiple strands of evidence indicate that the pathological changes of AD gradually accrue over a period of many years before the onset of symptoms [62,63]. Much of this research has been done on familial AD mutation carriers, where a variety of imaging abnormalities is observed during the presymptomatic phase of disease. These abnormalities include early amyloid accumulation [63-65], regional hypometabolism [63], atrophy [66,67], and alterations to functional connectivity [68] and tissue microstructure [66,69]. All of these studies are reviewed in more detail in [70].

A number of preclinical prevention trials for AD, either in planning or recruiting, are based on two major strategies in terms of population selection. The first strategy is to recruit participants who are known to be at increased genetic risk for AD – either because they carry an autosomal dominant mutation that causes AD or because they are a carrier of the ApoE ε4 allele. The Dominantly Inherited Alzheimer Network is an international biomarker study of families affected by familial AD with sites across the USA, Australia and Europe [71]. The Dominantly Inherited Alzheimer Network has launched a Trials Unit that is enrolling patients into a double-blind placebo-controlled trial where participants may receive either gantenerumab or solaneuzumab. Primary endpoints are changes in amyloid burden, as determined by PIB scans for the gantenerumab arm and by CSF amyloid-beta levels in the solaneuzumab arm. Secondary endpoints will involve further measures of amyloid, rates of brain atrophy and changes on FDG-PET imaging in key regions of interest, such as the precuneus. The Alzheimer’s Prevention Initiative, which follows a large Colombian kindred affected by the presenilin 1 gene PSEN1 E280A mutation [72], will begin treatment with crenezumab, another anti-amyloid monoclonal antibody. This trial has cognitive primary outcome measures but imaging changes (PIB, structural MRI and FDG-PET) will serve as secondary endpoints.

The second strategy is to recruit subjects that have high risk for AD according to the recent definition of preclinical AD. The A4 (Anti-Amyloid Treatment in Asymptomatic Alzheimer’s) study aims to enrol 1,000 cognitively normal individuals aged 65 to 85 with evidence of amyloid accumulation on amyloid PET imaging into a trial with solanezumab [73]. This trial also has cognitive primary endpoints but will assess changes on amyloid imaging, volumetric MRI and CSF as secondary outcome measures. All of the planned prevention trials will therefore collect multiple biomarkers, providing complementary information about the disease process and the potential effects of intervention, with the hope that these will inform the establishment of surrogate endpoints for prevention trials in the future [74].

### Imaging’s role in dementia trials: regulatory guidance

All of the trials reviewed in this article are attempting to collect substantial evidence for efficacy and safety, which is required to obtain regulatory approval (for example, by the US Food and Drug Administration). Historically, the criterion for achieving this approval is to meet the primary endpoint of two independent double-blind, placebo-controlled phase III trials.

Realising the urgent need for disease-modifying treatments, both the European Medicines Agency and the US Food and Drug Administration have provided guidance for drug development in AD and dementia [75,76]. While both advocate for a co-primary endpoint design using one cognitive and one functional (or global) endpoint, they also recognise that improvement on these endpoints alone could be a temporary, reversible effect that would not warrant a disease modification label for the drug. The current guidance from the US Food and Drug Administration states that they ‘are open to considering the argument that a positive biomarker result (generally included as a secondary outcome measure in a trial) in combination with a positive finding on a primary clinical outcome measure may support a claim of disease modification’ [75].

Imaging measures, which provide information on the underlying disease process, could be useful in providing evidence for disease modification. However, there is currently not enough evidence to validate any imaging biomarker as a surrogate biomarker and potential primary endpoint for a clinical trial [77]. There is also the risk of a circular logic: imaging biomarkers may provide key evidence of disease modification, but until we have a treatment that is truly disease modifying it is unclear what its effect on imaging biomarkers will be.

## Conclusions

Imaging is now used in clinical trials in AD at all stages of disease and drug development. MRI is a prerequisite safety outcome for some studies. The search for disease-modifying therapies will increasingly incorporate multiple imaging endpoints to assess alterations in molecular pathology (for example, amyloid and now tau imaging) and downstream effects of neurodegeneration on structure (for example, atrophy on MRI), function and connectivity (for example, FDG-PET, functional MRI and diffusion imaging). Moving to presymptomatic trials will increase the importance of imaging and biomarkers. Ultimately, it will only be with confirmation of a clinically useful disease modification effect that we will know the value of imaging endpoints.

## Additional file

Additional file 1:
**Is a table presenting combined results from the PubMed and ClinicalTrials.gov database searches.**

## Abbreviations

AD: Alzheimer’s disease
ApoE: Apolipoprotein E
ARIA: Amyloid-related imaging abnormalities
CSF: Cerebrospinal fluid
FDG: Fludeoxyglucose
MCI: Mild cognitive impairment
MRI: Magnetic resonance imaging
PET: Positron emission tomography
PIB: Pittsburg compound B
